# Decadal variability of extreme wave height representing storm severity in the northeast Atlantic and North Sea since the foundation of the Royal Society

**DOI:** 10.1098/rspa.2016.0376

**Published:** 2016-09

**Authors:** H. Santo, P. H. Taylor, R. Gibson

**Affiliations:** 1 Centre for Offshore Research & Engineering, Department of Civil and Environmental Engineering, National University of Singapore, Singapore, Republic of Singapore; 2 Department of Engineering Science, University of Oxford, Oxford, UK; 3 Offshore Consulting Group, London, UK

**Keywords:** extreme wave height, variability, correlation with NAO, reconstruction of extreme wave climate

## Abstract

Long-term estimation of extreme wave height remains a key challenge because of the short duration of available wave data, and also because of the possible impact of climate variability on ocean waves. Here, we analyse storm-based statistics to obtain estimates of extreme wave height at locations in the northeast Atlantic and North Sea using the NORA10 wave hindcast (1958–2011), and use a 5 year sliding window to examine temporal variability. The decadal variability is correlated to the North Atlantic oscillation and other atmospheric modes, using a six-term predictor model incorporating the climate indices and their Hilbert transforms. This allows reconstruction of the historic extreme climate back to 1661, using a combination of known and proxy climate indices. Significant decadal variability primarily driven by the North Atlantic oscillation is observed, and this should be considered for the long-term survivability of offshore structures and marine renewable energy devices. The analysis on wave climate reconstruction reveals that the variation of the mean, 99th percentile and extreme wave climates over decadal time scales for locations close to the dominant storm tracks in the open North Atlantic are comparable, whereas the wave climates for the rest of the locations including the North Sea are rather different.

## 1. Introduction

Extreme wave climate is important for the design of offshore structures, marine operations and coastal defences. Accurate estimation of such extremes requires long-term, good-quality measurements, which are rarely available, and hence this remains a key issue. Studies of the extreme wave climate have increased in number in recent years. Both global and regional estimates of extreme significant wave height (*H*_*s*_) values, as well as long-term trend analysis, have been produced [1–5]. Seasonal and interannual variability of extreme *H*_*s*_ values has been assessed, and associated with large-scale pressure anomalies or teleconnections, such as the North Atlantic oscillation (NAO) in the northeast Atlantic [6–9].

Are the properties of waves in the Atlantic over the last 50 years a reliable guide to what may happen in the next 50 or 100 years? We aim to tackle this question by seeking correlations between the variability of the extreme wave heights in the northeast Atlantic and the North Sea and large-scale atmospheric teleconnections. For this region, we include the NAO, and the next two modes, which are the East Atlantic (EA) pattern, and the Scandinavian pattern (SCA). The NAO measures variation in the Atlantic eddy-driven jet that controls the near-surface westerly winds [10], the EA pattern affects the position of the dominant North Atlantic storm track and jet streams [10] and the SCA pattern influences cyclone activity across Northern Europe and Eurasia [11]. Hence, a link between pressure anomalies and extreme wave climate is likely. The NAO and other modes are found to correlate well with mean wave climate [12], so we extend this approach to extreme waves. The temporal pattern of each mode is characterized by a climate index, values of which are obtainable from National Oceanic and Atmospheric Administration (NOAA) Climate Prediction Center (CPC; www.cpc.ncep.noaa.gov), and are based on the rotated empirical orthogonal function analysis by Barnston & Livezey [13]. The available indices cover January 1950 to the present.

Here we present extreme value analysis on storm severity by using a peaks-over-threshold (POT) technique to fit the upper tail of the data with an exponential distribution. The analysis is performed on a sliding window over 5 year blocks to produce temporal variability for correlation with the NAO and other modes. The results are confirmed by comparison with the decadal variation of the 99th percentile of *H*_*s*_ which requires no extrapolation. This study is a continuation of previous work by Taylor and co-workers [14,15] using earlier hindcast wave models, and Santo *et al*. [12], who looked at annual mean wave power variability using the same hindcast wave model.

## 2. Data and methods

**Figure 1.**
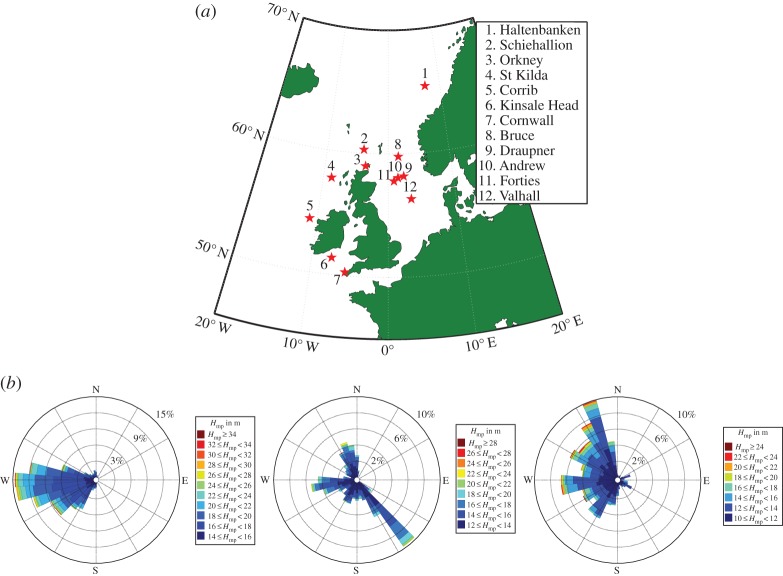
Geographic and wave information of the locations of the wave data. (*a*) Map of the locations, with points 1–7 in the northeast Atlantic, 8–12 in the North Sea. (*b*) The largest 1000 storm severity distribution in terms of the most probable maximum individual wave height (*H*_mp_) for St Kilda, Bruce and Valhall (points 4, 8 and 12, from left to right). The range circles show the frequency (in %) of *H*_mp_ occurrence normalized by the total occurrences for all directional bins of 10°. (Online version in colour.)

We examine hindcast wave data at 12 locations in the northeast Atlantic and the North Sea from 1958 to 2011, as shown in figure 1*a*. The hindcast data are taken from the Norwegian 10 km Reanalysis Archive (NORA10), for more information, see [16]. We also use measured buoy data for Haltenbanken from 1980–2000 (and Forties from 1974 to 1996 but this contains large gaps, so analysis of decadal variation of extreme values is not attempted). Both hindcast and measured wave data were sampled at 3 h intervals, and contain information such as date, time, significant wave height (*H*_*s*_), peak spectral wave period (*T*_*p*_), wind speed, wind and wave directions. For the extreme wave height analysis, *H*_*s*_ and *T*_*p*_ are used. Previous analysis of mean wave power variability using the same data had investigated the comparisons of hindcast/buoys in terms of the annual mean values at Haltenbanken and Forties, and in general, the agreement is reasonable [12].

Figure 1*b* shows directional wave rose distributions for the largest 1000 storms at three locations: St Kilda, Bruce and Valhall (points 4, 8 and 12, respectively), shown in terms of the most probable maximum individual wave height (*H*_mp_), which is a measure of storm severity (discussed in §2a). The wave direction for each *H*_mp_ is set to the wave direction of the peak *H*_*s*_ within each storm. The range circles show the frequency (in %) of *H*_mp_ occurrence normalized by the total occurrences for all directional bins of 10°. The wave rose distributions for the rest of the locations are provided in electronic supplementary material, figure S1.

The dominant large waves in the northeast Atlantic locations (points 1–7, including St Kilda) come from the west generated by storms in the open North Atlantic, and consistent with the dominant wave direction contributing to the mean wave power variability, see [12]. For the North Sea locations (points 8–11) except Valhall (point 12), the dominant large waves come from effectively two directional sectors (northwest and southeast), similar to the two dominant wave directions contributing to the mean wave power variability. It is also interesting to see that, at Bruce, waves propagate from the west through the Orkney–Shetland gap and from the north coming down the east coast of the Shetlands.

Northwesterly waves get weaker further south. Thus, one would expect a systematic decrease in the strength of the extreme waves moving south down the North Sea. There is a second contribution of waves from effectively the opposite direction (from the southeast), justifying partitioning the data on incoming wave direction. Curiously, Valhall is the only location in the North Sea that we have examined where the dominant large waves are from one directional sector (from north to southwest); there are smaller waves from the southeast sector but these are not shown as they are all below the POT-threshold.

### (a) Storm-based identification

Simple extreme value analysis methods require observations to be independent and identically distributed. Successive *H*_s_ values are strongly correlated as they are likely to be part of a single longer storm. Hence, storms are considered for long-term extrapolation instead. The entire (*H*_s_,*T*_p_) record is grouped into storms, which are effectively uncorrelated. Only winter storms are considered (from October to March every year). A single storm at a fixed location is assumed to last not more than 24 h, a reflection of the time scale for the motion of winter depressions in the northeast Atlantic and the North Sea [17]. Because the probability distribution of the upper tail of the individual wave height distribution for a storm is governed solely by the portion which has *H*_s_>0.8*H*_sMax_ [18], we use this threshold to define a single storm. We investigate the sensitivity of this threshold and the assumed upper limit for storm duration of 24 h on the 100 year return period estimates of *H*_mp_, and confirm that the results are robust, see electronic supplementary material, table S1 and figure S2.

Once all storms have been identified, the most probable largest individual wave in each storm (*H*_mp_) could be obtained following the method by Tromans & Vanderschuren [17], using convolution integrals. A simpler process to obtain *H*_mp_ by random sampling is presented as follows. For each (*H*_s_,*T*_p_) record in a storm, the corresponding zero crossing period of individual waves, *T*_z_, is estimated using *T*_z_=0.779*T*_p_ assuming the JONSWAP spectral shape, which gives an estimate of the number of waves (*N*_w_) in each 3 h sea-state [19,18]. Assuming a Rayleigh distribution to account for the intrasea-state and intrastorm variability of individual wave heights within a sea-state, the probability (*P*) that all *N*_w_ peaks are simultaneously less than a wave amplitude, *A*, can be expressed as

2.1 $$P={\left[1-\exp\left(-\frac{A^{2}}{2\sigma^{2}}\right)\right]}^{N_{w}},$$

where *σ*=*H*_s_/4 and *A* is obtained by randomly sampling the probability *P* between 0 and 1. This is performed for each *H*_s_ record (each 3 h sea-state) in a storm and $A_{\max}$ from all sea-states at any time during each storm is found. This sampling process is repeated 1000 times to estimate the histogram of all the possible $A_{\max}$ values, to which an empirical probability distribution function (PDF) derived from equation (2.1) can be fitted, expressed as

$$p=\frac{dP}{dA}=\frac{AN_{s}}{\sigma_{s}^{2}}\exp\left(-\frac{A^{2}}{2\sigma_{s}^{2}}\right){\left[1-\exp\left(-\frac{A^{2}}{2\sigma_{s}^{2}}\right)\right]}^{N_{s}-1}$$

with two-parameter optimization (*σ*_s_ and *N*_s_) performed by least-squares minimization. These parameters are those of an equivalent rectangular area storm, with *σ*_s_ being the intensity and *N*_s_ being the measure of storm duration. Other than using the fit to identify *H*_mp_, we make no other use of them here.

The peak value of the empirical PDF (or the mode) is the most probable maximum amplitude of the wave (*A*_mp_). For linear wave theory, the most probable maximum wave height within a storm is *H*_mp_=2×*A*_mp_. The use of *H*_mp_ accounts for the possibility that the largest wave may not always occur in the most severe 3 h sea-state and storms often last for more than 3 h. Hence, it is a more robust measure of storm severity than *H*_sMax_ [14,15,17]. While *H*_mp_ is a convenient parameter for estimating storm severity, in defining it, we are making a narrow-banded assumption about the individual waves in each sea-state. In addition, the 100 year return period estimate of individual wave height ($H_{\max}$) is likely to be 5–7% larger, thus *H*_mp_ should not be used as a design wave height. Nevertheless, because *H*_mp_ is less noisy than $H_{\max}$, it is a suitable parameter to correlate with the NAO.

As an alternative to the Rayleigh distribution, we could have used the distribution for wave height proposed by Forristall [20]. This has the drawback that wave height as conventionally defined is not a point-wise property of the record (being the sum of a crest height and following trough in time, with these separated by approximately half the wave period). Forristall's proposed distribution for the short-term statistics of wave crests [21] avoids this problem, but does not include the second-order difference term. In addition, crest elevation is a combination of a linear wave amplitude and a mostly double frequency sum contribution that makes crests and troughs different shapes. For structural reliability calculations, engineers usually start from the linear wave amplitude, or twice this being the local height of the wave envelope as used here; these being satisfactorily (and slightly conservatively) approximated using the simple Rayleigh distribution.

While we regard *H*_mp_ as an important storm-based parameter for engineering applications, we also present the results of extreme value analysis on the more commonly used *H*_sMax_ by using the same storm-based identification and taking the largest *H*_s_ record within each storm as *H*_sMax_ (one entry per storm). It is observed that *H*_mp_∼2×*H*_sMax_ for 100 and 1000 year return period estimates. We also present the results on the 99th percentile of the complete *H*_s_ record over all winters (from October to March every year) to produce an in-sample estimate for comparison with *H*_mp_ and *H*_sMax_. Unless otherwise specified, most of the results are presented in terms of 100 year return period of *H*_mp_, but we will show that the other parameters have very similar behaviour over decadal time scales.

### (b) Peaks-over-threshold technique

The identified storms are ordered in terms of *H*_mp_ (and *H*_sMax_) magnitude, and the POT technique is used for the long-term extrapolation. We seek a tail form for the cumulative density function (CDF) that fits the observations above a threshold (or exceedances). Formally, this is described by the generalized Pareto distribution (GPD) [22]. In general, there are three simple characteristic forms of the GPD described in terms of the thickness of the high tail: ‘fat’ tail, ‘thin’ tail and tail with an upper limit.

Here we assume that the exceedances fall into the class of ‘thin’ asymptotic tails with an exponential form (see eqn. 4.4 in [22]), with the CDF written as

2.2 $$P=1-\exp\left[-\left(\frac{H_{\mathrm{mp}}-H_{c}}{\alpha }\right)\right],$$

where *H*_*c*_ is the cut-off (threshold) value of *H*_mp_, and *α* is the scale parameter, to be obtained by the maximum-likelihood method.

We considered the possibility of a data transformation prior to estimating the exceedances (as discussed by [23]). This would be equivalent to replacing the exponential form with a Weibull fit commonly used by oceanographers (see eqn 6.8-4 in [18]), but for this particular case, the simple exponential fits well. Arguably, the exponential fit could be assumed across all the locations, because most of the storm-driven large waves are coming across the North Atlantic (from the wave rose distributions). It should be noted that most of the analysis is aimed at the 100 year (10^−2^ yr^−1^) wave. With 54 years of hindcast data from NORA10, little extrapolation is required. Therefore, we expect that almost any type of reasonable fit would yield comparable results.

## 3. Extreme wave height

First, we perform extreme value analysis using the entire 54 year hindcast storm record from 1958 to 2011, which assumes a stationary wave climate over 54 years, ignoring any long-term trends. Subsequently, to account for non-stationarity, we apply a sliding window over 5 year blocks assuming the data are stationary and identically distributed only within each 5 year period, and produce estimates of the temporal variability of the 100 year *H*_mp_ (10^−2^ risk per year) for correlation with the teleconnections. The stationarity assumption for the 54 year record is perhaps reasonable given that the long-term trend from the sliding window analysis is small, despite the large decadal variability that will be apparent later. It is worth stressing that stationarity is commonly assumed in standard offshore industry practice, unless intrarecord variability is specifically looked for. Using a POT method, the twin requirements of enough large values within the data and the sensitivity of the extrapolated values to threshold, so extreme value theory can be assumed, both drive the inclusion of as much data into a single analysis as possible.

**Figure 2.**
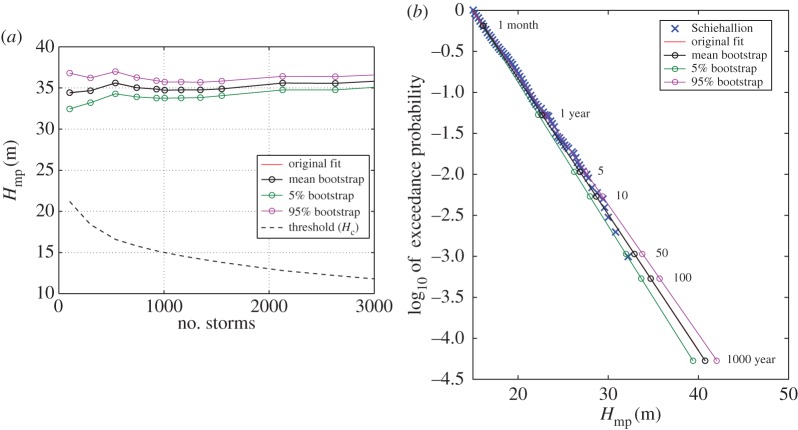
The 100 year most probable maximum wave height (*H*_mp_) for Schiehallion with an exponential fit. (*a*) Variation of the 100 year return period *H*_mp_ and the resulting *H*_c_ against the number of storms. (*b*) Long-term extrapolation using the exponential fit with parametric bootstrap for threshold *N*∼1000 largest storms. The circles from bottom up correspond to *H*_mp_ with a return period of one in 1000 years, 100 years, 50 years, 10 years, 5 years, 1 year and 1 month, respectively. The original fit lies beneath the mean bootstrap line. (Online version in colour.)

Because robust threshold behaviour is important for POT analysis, we investigate the sensitivity to threshold level using the entire 54 years of record. We use the number of storms (*N*) as a threshold for every location, hence the value of *H*_c_ will vary for each location depending on the storm severity over time. Figure 2*a* shows the variation in the predicted 100 year *H*_mp_ value at Schiehallion as the threshold *N* (and *H*_c_) for the fit is changed. The value of the 100 year *H*_mp_ is reasonably stable around *N*∼1000 largest storms, and this generally holds elsewhere, suggesting the choice of threshold level is robust. A threshold level of *N*∼1000 storms corresponds to approximately 20 storms per winter, roughly one to two per week.

Figure 2*b* shows the long-term fit of the *H*_mp_ for Schiehallion. Every data point represents an individual storm severity (*H*_mp_) from the hindcast, and all these lie within or close to the envelope, or trumpet (estimated 5–95% confidence interval), which is obtained by a parametric bootstrap. A very similar trumpet is also obtainable by non-parametric bootstrap (by sampling from the data itself). The mean of the bootstrap fits very closely to the original data fit, and the resultant trumpet is close to symmetric around the mean value, suggesting that the analysis is robust. Generally, the variation of the width of the trumpet at the 5–95% level for the 100 year *H*_mp_ is ∼7% of the estimated mean value.

The *H*_mp_ fits for the rest of the locations are summarized in table 1 and plotted in electronic supplementary material, figures S3 and S4. The ratio of the 1000 to 100 year *H*_mp_ (a measure of the slope of the long-term extrapolation) is about 1.17–1.18, consistent for all locations. In general, for the open North Atlantic locations, St Kilda has the most severe extreme wave height values, followed by Corrib, Schiehallion and Haltenbanken. For the North Sea locations, the extreme wave heights decrease slightly from north to south, consistent with the largest *H*_sMax_ values in the hindcast and with the wave height rose distributions in §2.

**Table 1.** Summary of the results of the 100 and 1000 year return period fits to *H*_sMax_ and *H*_mp_ and correlation of the 100 year return period *H*_mp_ with the teleconnections for all locations. The values in brackets [..] denote the return period.

	for the entire data					for the 5 year sliding window					
	*H*_sMax_ (m)			*H*_mp_ (m)		three-term model			six-term model		
location	*[54]*	*[100]*	*[1000]*	*[100]*	*[1000]*	*R*^2^	*R*^2^^*a*^	*R*^2^^*b*^	*R*^2^	*R*^2^^*a*^	*R*^2^^*b*^
for the period of available measurement (1980–2000) at Haltenbaken											
measured	14.0	17.0	20.0	33.0	38.7	0.69	0.75	—	0.92	0.99	—
hindcast	14.2	17.1	20.1	33.8	39.8	0.73	0.90	—	0.90	0.99	—
for the entire period (1958–2011) of hindcast data											
for the open North Atlantic locations											
1. Haltenbanken	17.0	16.7	19.7	33.0	38.8	0.60	0.74	0.57	0.67	0.79	0.62
2. Schiehallion	16.6	17.9	21.1	34.7	40.8	0.64	0.79	0.64	0.71	0.86	0.68
3. Orkney	15.3	16.2	19.2	31.4	37.1	0.54	0.73	0.54	0.62	0.86	0.62
4. St Kilda	17.7	18.6	21.8	36.3	42.6	0.45	0.51	0.44	0.54	0.69	0.52
5. Corrib	18.2	17.9	21.0	34.9	40.9	0.24	0.28	0.11	0.40	0.60	0.24
6. Kinsale Head	12.6	13.0	15.3	25.6	30.1	0.27	0.55	0.21	0.40	0.64	0.28
7. Cornwall	11.6	11.8	14.0	23.1	27.4	0.13	0.21	0.13	0.26	0.46	0.20
for the North Sea locations											
8. Bruce	13.4	14.4	16.9	28.5	33.3	0.13	0.32	—	0.20	0.35	—
9. Draupner	13.4	13.8	16.2	27.3	32.0	0.12	0.32	—	0.23	0.40	—
10. Andrew	13.4	13.8	16.2	27.3	32.0	0.18	0.39	—	0.30	0.45	—
11. Forties	12.8	13.5	15.9	26.6	31.3	0.17	0.26	—	0.21	0.27	—
12. Valhall	12.0	13.1	15.5	25.8	30.3	0.48	0.74	—	0.63	0.87	—

^*a*^Correlation between the 5 year sliding window signals and the teleconnections in terms of 5 year moving average.

^*b*^Correlation between the 5 year sliding window signals and the teleconnections after removing the smallest contribution from an atmospheric mode for locations in the open North Atlantic. The smallest contribution for almost all locations is from the EA, except locations 2 and 3 where this is the SCA. The value is to be compared with the previous *R*^2^ value using all three indices for the three-term model plus the Hilbert transforms for the six-term model.

**Figure 3.**
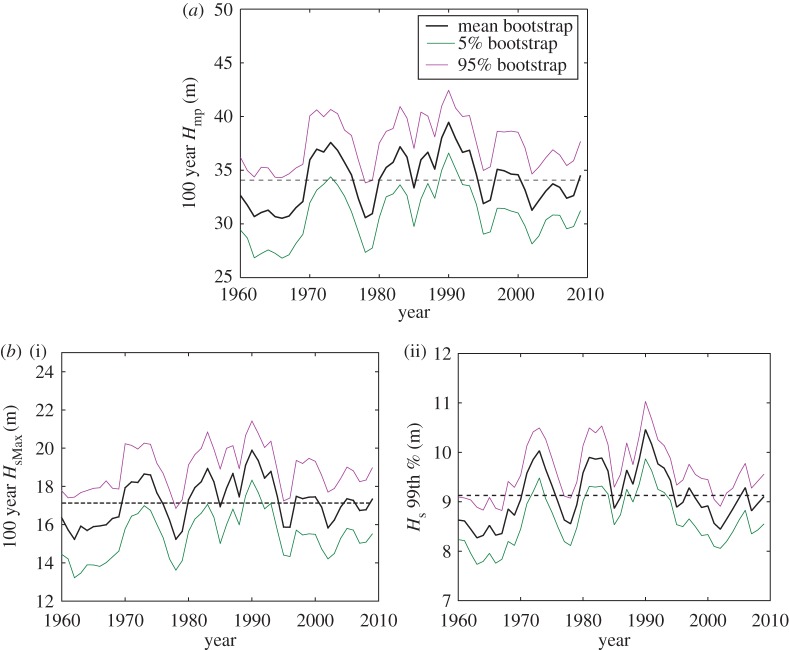
Variation of the 5 year sliding window for Schiehallion with an exponential fit. Dashed line is the long-term mean value of the sliding window. (*a*) In terms of the 100 year return period *H*_mp_. (*b*) In terms of the 100 year return period *H*_sMax_ (i) and the 99th percentile of *H*_s_ (ii). (Online version in colour.)

Figure 3*a* shows the 5 year sliding window of the 100 year *H*_mp_ for Schiehallion. The definition of the storm year is based on the year of December of the third winter, and two winters either side. With *N*∼1000 storms over 54 years, a 5 year sliding block contains ∼100 storms (on average), which we judge sufficient for extreme value analysis. The signal is dominated by significant decadal variability with no obvious long-term trend, and it is this decadal variability that will be correlated with the teleconnections. Also shown is the long-term mean value (dashed line) of the 5 year based 100 year value, which here for Schiehallion is 34.08 m, only 2% different from the value for the entire 54 years storm record (34.73 m, table 1). Thus, the long-term trend of the data is small, and this also holds for all locations. The degree of the temporal variability for all location ranges from 5% to 8% in terms of coefficient of variation.

On the same figure, the estimated 5–95% confidence interval represents the variability by random sampling within each 5 year block. Given that the long-term mean value is within the broad 5–95% confidence interval, is the time-varying signal driven by real large-scale atmospheric variability, or is it just statistical noise from a long-term constant distribution? In this paper, we present evidence that this variation is real because of the strong correlation with the NAO and other modes.

Figure 3*b* shows the 5 year sliding window of the 100 year *H*_sMax_ (i) and the 99th percentile of *H*_s_ (ii) for Schiehallion. The sliding window results for the 100 year *H*_sMax_ is obtained using the same procedure as for the 100 year *H*_mp_, whereas for the 99th percentile of *H*_s_, the value of the 99th percentile is obtained on an annual basis over 54 years, and a 5 year moving average is run across the signal to produce comparable 5 year sliding window results. In general, the decadal variability in both the 100 year *H*_sMax_ and the 99th percentile of *H*_s_ is similar to that of the 100 year *H*_mp_. However, there is some slight difference in the year-to-year variation of the 99th percentile of *H*_s_ when compared with the other two results, which is likely owing to finite size sample variability in the POT analysis. This is also evident from the width of the estimated 5–95% confidence interval which is narrower for the in-sample estimate of the 99th percentile of *H*_s_. In addition, it is worth noting that *H*_sMax_∼2× 99th percentile of *H*_s_, so we are looking at a different part of the distributions which are treated in a rather different manner, hence the level of agreement in the ‘wiggles’ is encouraging. We consider that the similarity in the decadal variability of the POT results with that of the in-sample estimate demonstrates the robustness of our extreme value analysis.

**Figure 4.**
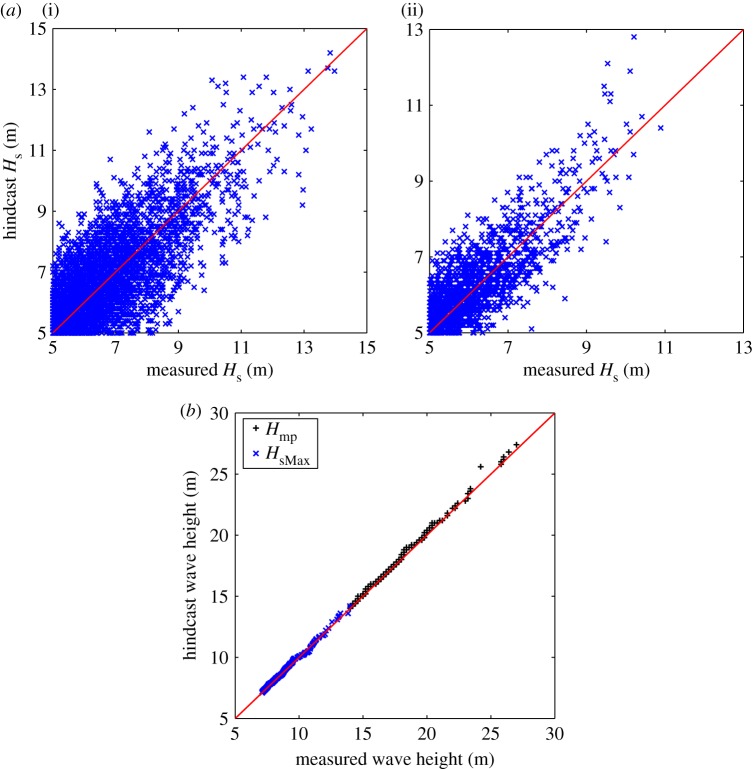
Model/buoy comparisons. (*a*) In terms of individual *H*_s_ for Haltenbanken (i) and Forties (ii). (*b*) In terms of ranked order storm-by-storm based on the largest 450 wave heights for Haltenbanken. (Online version in colour.)

**Figure 5.**
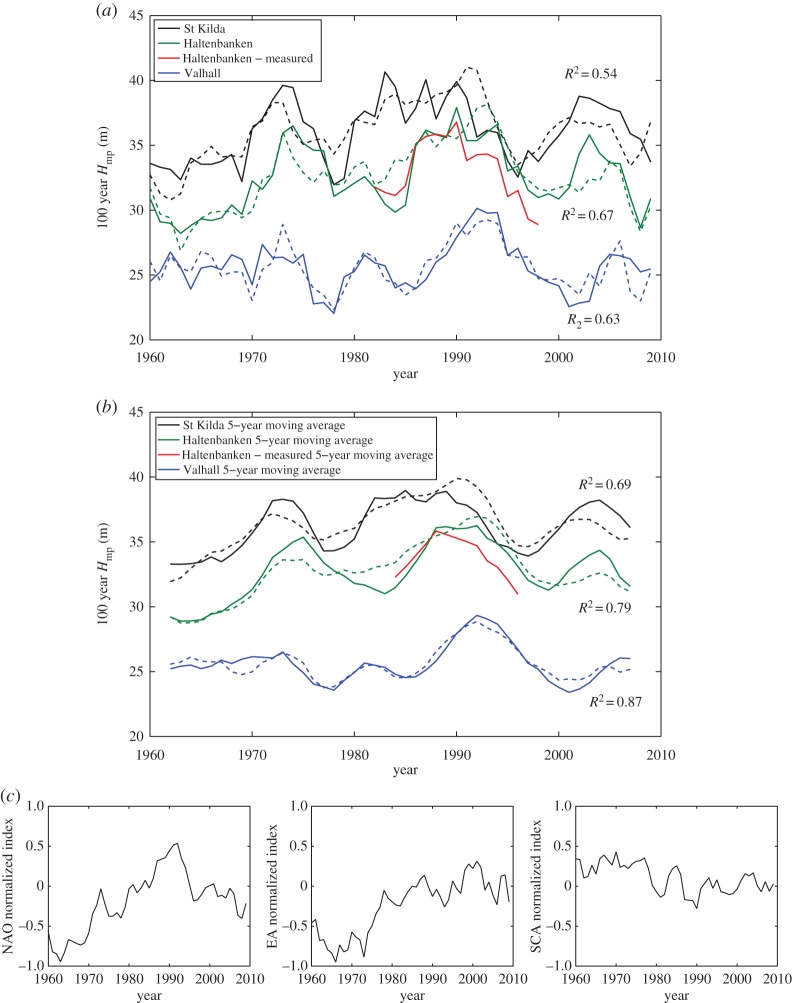
(*a*) Five yearsliding window results in terms of 100 year *H*_mp_ (solid lines) and the teleconnection-based prediction (dashed lines), for St Kilda (black line), Haltenbanken (green line) and Valhall (blue line), and with measured buoy results for Haltenbanken (red line). (*b*) With an imposed 5 year moving average on each signal. (*c*) Variation of the 5 year moving averages of the atmospheric indices. (Online version in colour.)

Hindcast/buoy comparison in terms of individual *H*_s_ above 5 m is performed for Haltenbanken and Forties. Normalized root-mean-squared error is used to quantify the comparison: 15% for Haltenbanken and 11% for Forties with no bias. For storm-by-storm analysis, the comparison is performed for Haltenbanken for 21 years, from 1980 to 2000, with *N*∼450 storms to yield a consistent ∼100 storms per 5 year block on average. We compare the largest 450 *H*_sMax_ and *H*_mp_ values (ranked order storm), and the agreement is satisfactory (figure 4). The comparison of the 100 year and 1000 year extreme values are close (table 1). For a 5 year sliding window on the same period of 21 years, the temporal variation of the 100 year values are similarly close, with *R*^2^=0.75 (figure 5, buoy-based results in red), supporting the use of the hindcast wave data.

## 4. Correlation with the teleconnections

Following the method of correlating the annual mean wave power with the teleconnections described in [12], we correlate the 5 year sliding window estimates of the 100 year *H*_mp_ with the NAO, the EA and the SCA using the same winter average of the climate indices (obtained by averaging six month values of monthly teleconnection indices centred around the middle of January) and the same form of the predictor model, which is based on linear regression. We then compare the performance of this three-term predictor model with an extended six-term predictor model, which incorporates both the three climate indices and their Hilbert transforms of the indices (which represent 90° phase shifted versions and are related to the time derivative of the indices without amplification of high-frequency noise) [24,25].

The form of the six-term predictor model is expressed as

$$\begin{array}{rl} H_{\mathrm{mp}}(t) & ={\bar{H\;}\;\;}_{\mathrm{mp}}\times [1+b_{1}(\mathrm{NAO}(t)-\bar{\mathrm{NAO}})+c_{1}(\mathrm{EA}(t)-\bar{\mathrm{EA}})+d_{1}(\mathrm{SCA}(t)-\bar{\mathrm{SCA}})\\ & \;+b_{2}({\mathrm{NAO}}_{H}(t)-{\bar{\mathrm{NAO}}}_{H})+c_{2}({\mathrm{EA}}_{H}(t)-{\bar{\mathrm{EA}}}_{H})+d_{2}({\mathrm{SCA}}_{H}(t)-{\bar{\mathrm{SCA}}}_{H})], \end{array}$$

where ${\bar{H\;}\;\;}_{\mathrm{mp}}$ is the long-term mean of *H*_mp_ over the period of available hindcast data, *NAO*(*t*) is the temporal winter average of the NAO index, $\bar{\mathrm{NAO}}$ is the long-term mean of the *NAO*(*t*), likewise for *EA*(*t*) and *SCA*(*t*). The subscript *H* represents the Hilbert transform of the indices. *b*, *c* and *d* are non-dimensionalized constants resulting from the variance minimization; these reflect the relative importance of the NAO, the EA and the SCA signals (subscripts 1), and their Hilbert transform counterparts (subscripts 2) in describing variability of extreme wave height. For *b*_2_=*c*_2_=*d*_2_=0, the above form reduces to the three-term predictor model. The predictor model is trained over the period of available hindcast data.

The Hilbert transform is performed on each index running from 1950 to present, which has been patched at each end of the signal with a scaled autocorrelation of the index to minimize end effects. Arguably, we are not over-fitting the model as the additional three index signals are derived analytically from the original three indices (a Hilbert transform of a signal is orthogonal to the original signal). For both predictor models, we impose a low-pass filter of 5 years to all climate indices and Hilbert-transformed indices (moving average), to be consistent with our 5 year sliding window analysis.

The correlation results of the 5 year sliding window for all the locations are summarized in table 1. In general, the correlations at all locations are improved with the six-term predictor model compared with the three-term model, demonstrating the ability of the six-term model in capturing more of the short-term variability within the training period (50 years). More importantly, the improved results are more consistent, i.e. we obtain reasonable correlations with the NAO and other modes uniformly across the locations in the open North Atlantic with the six-term model. Additional information regarding the optimized non-dimensionalized constants is provided in electronic supplementary material, tables S2 and S3. In general, the NAO has the largest contribution when the *R*^2^ is significant (locations 1–4), with contributions being measured in terms of the relative strength of the non-dimensionalized constants in each predictor model. However, some of the correlation results look peculiar, such as from locations 5–7 (going southwards) where the SCA is seen to have the most dominant contribution over the NAO and the EA.

Figure 5*a* illustrates, using the six-term predictor model, the correlation result of the 5 year sliding window for St Kilda, Haltenbanken and Valhall in terms of the one in 100 year *H*_mp_ (*R*^2^=0.54−0.67). We note the similarity of the extreme *H*_mp_ and the predictor model over time scales longer than 5 years. This becomes evident when a 5 year moving average is run across each signal without doing any further computation, and the correlation is improved (*R*^2^=0.69−0.87), see figure 5*b* and the second column under each predictor model in table 1. It is likely that the long-term variability beyond 5 years can be represented well by the NAO and other modes, but for intradecadal variability over less than 5 years, finite size sample variability in the POT analysis introduces significant numerical noise. The correlation results for the rest of the locations are provided in electronic supplementary material, figures S5 and S6 for the six-term model. Analysis of the residual term for each comparison reveals no significant autocorrelation structure beyond a 5 year lag. The correlation result of the one in 100 year *H*_sMax_ for all three locations is similar to that of the *H*_mp_ for the same return period, as evident from the similarity in the temporal variation of the 5 year sliding window result of both as shown previously in figure 3. The correlation result of the 99th percentile of *H*_s_ is improved for Haltenbanken and St Kilda with *R*^2^=0.83 and 0.71, respectively, whereas the correlation for Valhall is similar to that of *H*_mp_ with *R*^2^=0.65.

Figure 5*c* shows the variation of the 5 year moving average of the three atmospheric indices used in the predictor model (the associated Hilbert transforms are not shown). One can observe close resemblance of the variation between the NAO and the one in 100 year *H*_mp_ at the locations considered in figure 5*a*, likewise for the variation in the EA, whereas it is less obvious for the SCA. When the NAO is in positive phase (or less negative), the extreme wave activity is observed to increase accordingly, such as for the periods between 1970–1980 and in the 1990s, and vice versa when the NAO is in negative phase (or less positive). The strong relationship between the phase of the NAO and the wave variability, consistent with [26], is supported by the general observation that the positive phase of the NAO (and in combination with other atmospheric modes) is associated with warm and wet winters (plenty of large waves) in Northern Europe, while the negative phase is associated with cold and dry winters (fewer large waves).

As suggested by one of the reviewers, the question of whether the predominantly North Sea predictors EA and the SCA would benefit reconstruction of waves in the North Atlantic, we investigate the relative importance by dropping out the least significant atmospheric mode which is either the EA or the SCA. The effect on the *R*^2^ is assessed, which is shown in table 1 in the third column for each predictor model. The smallest contribution is from the EA for almost all locations, except locations 2–3 where the SCA has the least contribution. In general, the reduction in the *R*^2^ (or Δ*R*^2^) is larger for the six-term model compared with the three-term model for all locations. From the six-term model, we observe that all three atmospheric indices are significant (Δ*R*^2^ = 5–67%), so should be included in the predictor model except for locations 2–4 (Δ*R*^2^<5%). Hence, we retain all three indices (and the associated Hilbert transforms) for the subsequent analysis.

From table 1, reasonable correlation (*R*^2^>0.6) on the extreme wave height is obtained for the open North Atlantic locations (points 1–6) and Valhall (point 12), but the correlations for the rest of the locations (point 7–11) are rather weak. The correlation for Bruce and other locations in the North Sea is weak owing to the existence of two (or more) directional sectors. We split the omnidirectional waves into two directional sectors based on incoming wave directions, and investigate the temporal behaviour of each sector using the same methodology. The waves at Bruce (point 8), for instance, are split into the northwest (230°–50°) and southeast (50–230°) sectors, where 0° is pointing to the north and 90° to the east. Dramatically improved correlation in terms of 5 year moving average is obtained for the northwest sector (*R*^2^=0.90) when compared with the omnidirectional signal (*R*^2^=0.35); however, the southeast sector remains mostly unexplained by the NAO and other modes (*R*^2^=0.41). The correlation results for locations in the North Sea are summarized in table 2, and plotted in electronic supplementary material, figure S7. The correlation for Cornwall is weak perhaps because of the close proximity to shore and long distance sheltering effects from Ireland.

**Table 2.** Correlation results for the North Sea locations with angle partitioning.

	three-term model		six-term model	
location and direction	*R*^2^	*R*^2^^*a*^	*R*^2^	*R*^2^^*a*^
Bruce				
omnidirectional	0.13	0.32	0.20	0.35
northwest	0.67	0.80	0.73	0.90
southeast	0.11	0.11	0.28	0.41
Draupner				
omnidirectional	0.12	0.32	0.23	0.40
northwest	0.45	0.75	0.45	0.77
southeast	0.10	0.22	0.15	0.24
Andrew				
omnidirectional	0.18	0.39	0.30	0.45
northwest	0.29	0.48	0.34	0.56
southeast	0.02	0.03	0.08	0.09
Forties				
omnidirectional	0.10	0.26	0.21	0.27
northwest	0.40	0.58	0.45	0.69
southeast	0.06	0.16	0.13	0.23

^*a*^Correlation between the 5 year sliding window signals and the teleconnections in terms of 5 year moving average.

Overall, with the six-term predictor model, we observe that the westerly extreme wave heights at all locations are significantly correlated to the NAO and other modes (*R*^2^=0.6–0.9). In particular, points 1–4 and 8 have the strongest correlation with the NAO, apart from point 12. Interestingly, these locations are in close proximity to the dominant storm tracks. Because the NAO is known to be correlated with movements of the storm tracks, and latitude shifts of storm tracks are associated with changes of the largest waves in the North Atlantic [27], a strong relationship between the NAO and the extreme wave climate at these locations is expected.

## 5. Reconstructions of extreme wave climates

Significant correlation between the extreme wave climate and the dominant teleconnections over the period of the available hindcast data allows reconstruction of historic extreme wave climate, under the assumption that such relations hold into the past. We use the same proxy climate indices from 1659 to 1998 as used previously in [12]. The proxy indices were obtained by regressing the known indices from the CPC with the historical reconstructed monthly 500 mbar pressure maps computed by Luterbacher *et al*. [28], over the overlapping period from 1950 to 1998, see [12] for more details. Because our reconstruction analysis relies solely on the pressure data reconstructed by Luterbacher *et al.* [28], we stress that these winter atmospheric data fields are assumed to be correct over the period of the reconstruction. Subsequently, we apply the same technique to obtain the Hilbert transformed versions of the proxy indices. However, these contain unresolved long time-scale trends longer than the training period, which hamper our effort to reconstruct the wave climate based on the six-term model. To obtain the long time scale in our reconstruction, we make the following assumption.

**Figure 6.**
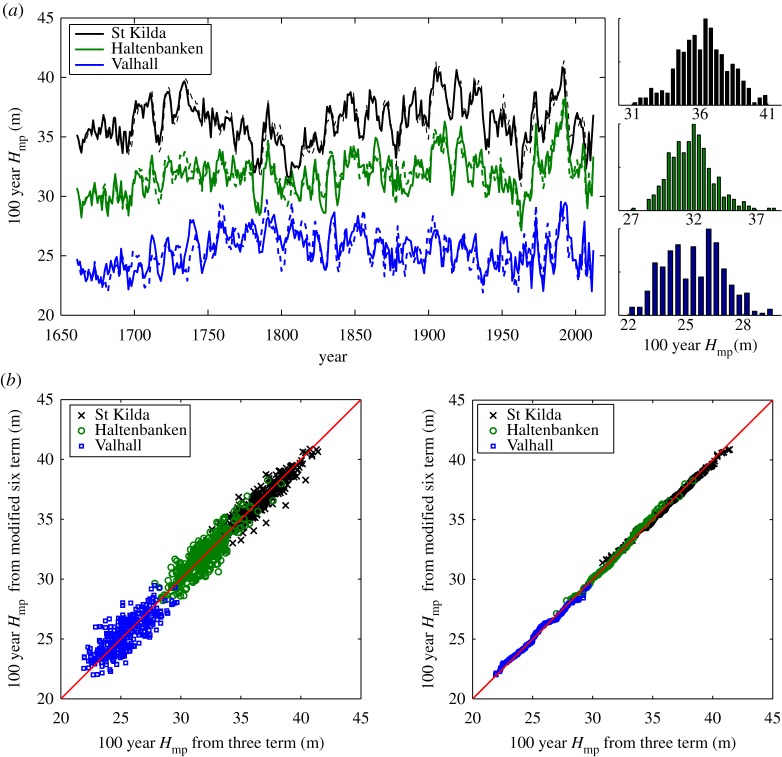
(*a*) Reconstruction of the 100 year *H*_mp_ climate from 1661 to 2012 using the modified six-term predictor model (solid lines) and the three-term model (dashed lines) for St Kilda (black lines), Haltenbanken (green lines) and Valhall (blue lines). The predictor uses proxy indices from 1661 to 1961 and known indices from 1962 to 2012. Also shown is the normalized histogram of the reconstructed 100 year *H*_mp_ for each location. (*b*) Comparison of the reconstructed 100 year *H*_mp_ between thesix-term predictor model and the three-term model for all three locations over the period from 1661 to 2012. At left figure, each point corresponds to a single reconstructed 5 year block. Right figure shows in terms of ranked order comparison. (Online version in colour.)

By assuming the long time-scale variation is sufficiently captured by three (proxy) climate indices, we can perform the reconstruction using the three-term model as previously applied in [12]. Based on the same assumption, we can also perform the reconstruction using a modified six-term model, containing the short time-scale fluctuations (less than 50 years) from the six-term model with the long time-scale variation (more than 50 years) described by the three-term model. Figure 6*a* shows the reconstructed one in 100 year *H*_mp_ at St Kilda, Haltenbanken and Valhall from 1661 to 2012, obtained by a combination of proxy indices from 1661 to 1961 and known indices from 1962 to 2012, using the modified six-term predictor model (solid lines) and the three-term model (dashed lines). Overall, the reconstructed results are similar for each location, and during the training period, the agreement between the two models is very close. Also shown on the same figure is the normalized histogram of the reconstruction for each location, these are approximately normally distributed. When the reconstructed results are plotted against each other over the period of reconstruction, the similarity between the two models becomes more evident, demonstrating the robustness of the two models in capturing the short time-scale fluctuations, as shown in figure 6*b*. However, because the six-term model is able to explain the short-term variability better than the three-term, the reconstruction from the modified six-term model should be more reliable.

It is worth remarking that Luterbacher's earlier data were generally based on terrestrial observations and this represents the marine region less well [29]. It was only from approximately 1850s onward that marine data well into the Atlantic were included. Thus, there has to be uncertainty in the reconstructed results in particular the long-term trend prior to the 1850s, and hence our reconstructions back then may not be as reliable as more recently. From figure 6*a*, we observe that in the 1990s the extreme wave height reaches the highest level ever seen (compared with the historic record) which aligns with the strongly positive phase of the NAO, and in the period from 1960 to 1990, there is a faster increase rate of the extremes for all locations. Also, there appear to be long-term trends at Haltenbanken and Valhall that may be statistically significant but these are influenced by apparently low activity prior to 1850s and by the recent heightened activity during the early 1990s.

**Figure 7.**
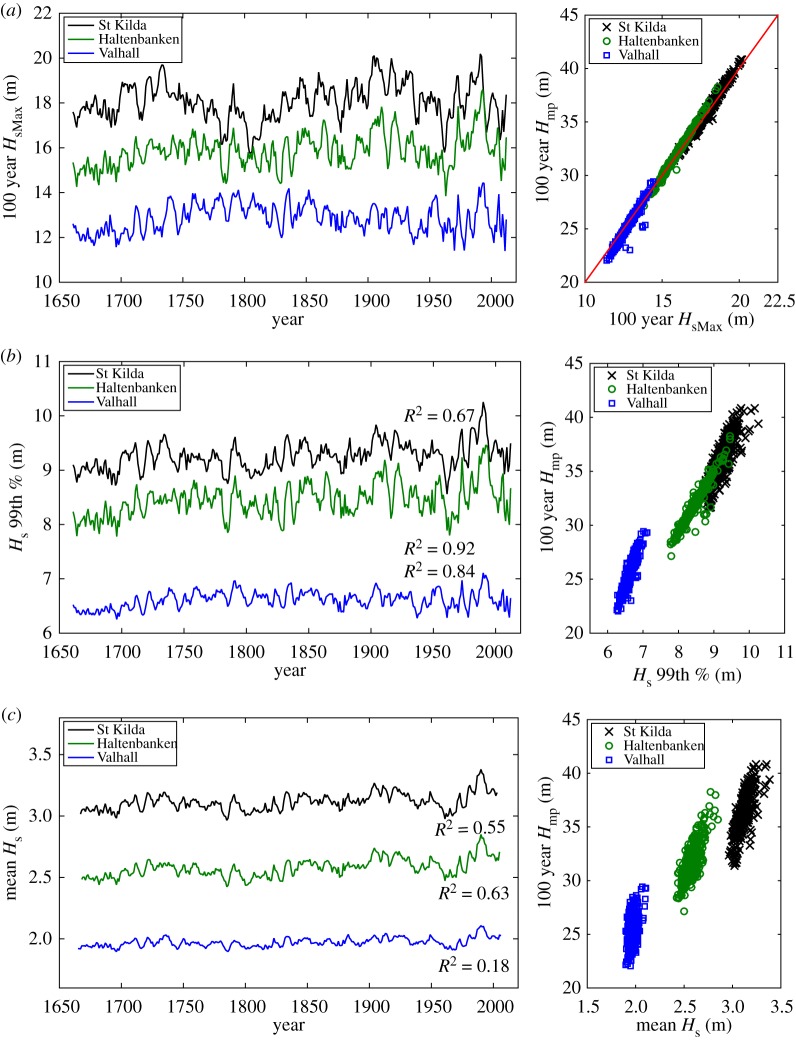
Left-hand side panels show the reconstruction of the (*a*) 100 year *H*_sMax_, (*b*) 99th percentile of *H*_s_ and (*c*) mean *H*_c_ climate, using the modified six-term predictor model for St Kilda (black lines), Haltenbanken (green lines) and Valhall (blue lines). The length of the reconstruction for both (*a*) and (*b*) is from 1661–2012, whereas that for (*c*) is from 1668 to 2005. The*R*^2^ values show the similarity in the temporal structure of the signal relative to one in 100 year *H*_mp_. Right-hand side panels show comparison of the reconstructed signal and the one in 100 year *H*_mp_. (Online version in colour.)

Using the same reconstruction analysis, figure 7*a* shows the reconstructed one in 100 year *H*_sMax_ for all three locations, using the modified six-term predictor model. It can be seen that the fluctuations at all time scales are very similar to those of the reconstructed one in 100 year *H*_mp_, which becomes more evident when the reconstructed results of *H*_sMax_ and *H*_mp_ are plotted against each other over the period of reconstruction. The solid red line is a 2:1 line showing the relative magnitude of *H*_mp_∼2×*H*_sMax_. This is in contrast to the usual estimate of *H*_mp_=1.86×*H*_sMax_ assuming a 3 h sea-state with *N*∼1000 waves. The factor of ∼2 implies storms last longer than 3 h, perhaps 3× as long on average, with *N*∼3000 waves. This is one indication that *H*_mp_ is a more robust measure of storm severity than *H*_sMax_ [14,15,17].

Figure 7*b* shows the reconstructed 99th percentile of *H*_s_ for the same locations using the modified six-term predictor model. It can be seen that the variability of the reconstructed 99th percentile of *H*_s_ is similar to that of the 100 year *H*_mp_, reflected by the *R*^2^ measured relative to the 100 year *H*_mp_. The close similarity between the two reconstructions demonstrates the robustness of our extreme value analysis, given that the 99th percentile requires no extrapolation and hence it is more robust measure of extremes (though it is not directly informative in a return period or reliability framework for offshore structural design for example). It should also be noted that these values of the 99th percentile of *H*_s_ are substantially smaller than any of the threshold *H*_mp_ values used for the POT analysis, hence we are observing not just teleconnections influencing the extremes, but also more values further down into the bulk of the distributions.

It is also of interest to reconstruct mean wave climate, represented by annual mean *H*_s_ for comparison with the reconstructed extreme wave climate. For such climates, we apply similar correlation methodology described in [12] using the three-term predictor model (with additional high-pass filtering applied to the EA and the SCA indices), and the six-term predictor model as introduced in this paper (without any filtering). The reconstruction of the mean *H*_s_ is then performed using the modified six-term predictor model based on the same assumption, and a 5 year moving average is run across the reconstructed signal. The reconstructed mean *H*_s_ for the same locations is shown in the same figure 7*c*. It is worth noting that because of the filtering applied in the three-term model for mean *H*_s_, the length of the reconstructed mean *H*_s_ (from 1668 to 2005) is slightly shorter than that of the one in 100 year *H*_mp_. The long-term mean and standard deviation of the reconstructions for all locations are provided in table 3.

**Table 3.** Summary of the results of the reconstruction of extreme and mean wave climates at all locations.

location	${\bar{H}}_{\mathrm{mp}-100}{\;}^{a}$ (m)	s.d.^*a*^ (m)	${\bar{H_{s}}}^{b}$ (m)	s.d.^*b*^ (m)	*R*^2^^*c*^
for the open North Atlantic locations				
1. Haltenbanken	31.8	1.73	2.58	0.07	0.63
2. Schiehallion	34.6	1.95	3.00	0.08	0.54
3. Orkney	31.5	1.14	2.26	0.07	0.32
4. St Kilda	36.2	1.88	3.11	0.07	0.55
5. Corrib	32.8	1.86	3.07	0.05	0.14
6. Kinsale Head	23.5	1.69	1.96	0.04	0.36
7. Cornwall	21.0	1.88	1.77	0.02	0.05
for the North Sea locations				
8. Bruce	28.8	1.15	2.40	0.05	0.14
9. Draupner	27.7	1.19	2.22	0.04	0.20
10. Andrew	27.3	1.35	2.17	0.04	0.27
11. Forties	27.2	1.19	2.11	0.03	0.09
12. Valhall	25.4	1.52	1.97	0.04	0.18

^*a*^Long-term mean and standard deviation of the reconstructed 100 year *H*_mp_.

^*b*^Long-term mean and standard deviation of the reconstructed annual mean *H*_s_.

^*c*^Correlation between the reconstructed 100 year *H*_mp_ and the reconstructed mean *H*_s_, both on a 5 year sliding window from 1668 to 2005.

From the wave climate reconstruction, similar temporal variation is observed between the reconstructed extreme, 99th percentile and mean wave climate for most of the open North Atlantic locations (locations 1–4), over the same 5 year sliding window. In contrast, the temporal structures between the mean and extreme wave climates are different for the North Sea and the rest of the locations. The similarity is measured in terms of *R*^2^ relative to the 100 year *H*_mp_, shown in the same figure and summarized in table 3 for all locations. Thus, the temporal variations in the extreme, 99th percentile and mean wave climates in these open North Atlantic locations are comparable, all these measures of the wave climates are influenced by the NAO because of the close proximity to the dominant storm tracks. For the rest of the locations including the North Sea, the extreme and mean wave climates are rather different: the mean wave climate is correlated to the NAO, but there is less evidence for the extreme wave climate. Also on the same figure, it is worth noting that the variation in the 99th percentile of *H*_s_ is closer to that of the 100 year *H*_mp_ compared with the mean *H*_s_, demonstrating that the use of the 99th percentile as an in-sample estimate of extremes is a reasonable approach.

**Figure 8.**
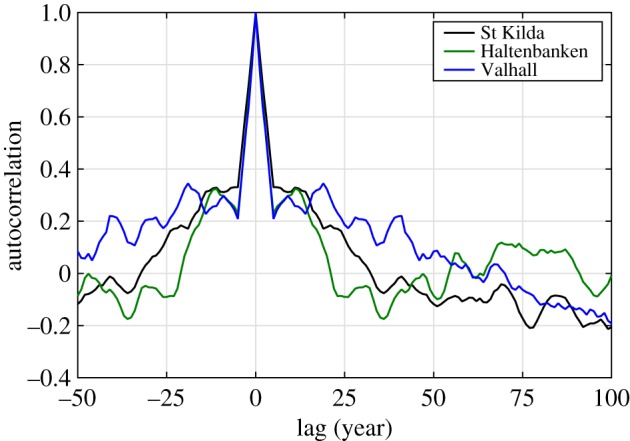
Autocorrelation structure of the reconstructed 100 year *H*_mp_ for all three locations as a function of year lag. (Online version in colour.)

Overall, our reconstructions show an unusual increasing trend in both extreme and mean wave climates during the period from the 1960s to 1990s. This is consistent with [31,30], who reported an increasing trend in mean wave climate in this region, and [2], who conducted extreme value analysis on *H*_sMax_ for locations close to Valhall, Bruce and Haltenbanken. However, our long time-scale analysis implies that the increasing trend during that period was strongly influenced by the variability of the ocean–atmosphere system driven mostly by the NAO. Our reconstruction seems to suggest that the wave model and wave observations of both *H*_sMax_ and *H*_mp_ during the strongly positive phase of the NAO in the early 1990s should be satisfactory for determining design wave specifications for coastal and offshore structures going forward, though the observations from around 1900 would be equally applicable. In general, large variations in the extreme wave climate are observed, which should be accounted for in any long-term survivability analysis for coastal, marine and offshore activities over time scales of several decades. Unfortunately, analysis of the autocorrelation structure suggests that there is little internal predictability, as shown in figure 8. A rapid drop of the predictability is observed within 5 years. Nevertheless, whether the next winter is rough or less rough might be important for offshore operations (a drilling campaign for example) as opposed to platform survivability.

## 6. Conclusion

Long-term extrapolation of extreme wave height based on hindcast wave data is presented, using a POT technique for locations in the northeast Atlantic and the North Sea. The estimates of the extreme wave height using a simple exponential fit to the entire 54 years of record are consistent with the storm severity each location experienced. We present our estimates based on the most probable maximum individual wave height in a storm or *H*_mp_, which is a measure of storm severity. We compare the results of the one in 100 year *H*_mp_ with the one in 100 year *H*_sMax_ as well as the in-sample 99th percentile of *H*_s_, and conclude that our estimate of *H*_mp_ is robust. To account for possible non-stationarity in extreme value analysis, we perform 5 year sliding window analysis to produce variability of the estimated extreme wave heights in time, which we then correlate with the NAO and other atmospheric modes. Improved correlation is obtained using the six-term predictor model compared with the three-term model during the period of available data, with the six-term model including both the climate indices and their Hilbert transforms. The long-term variability less than 50 years (the length of the wave hindcast) is correlated to the decadal structures of the NAO and other modes, whereas some of the intradecadal fluctuation over less than 5 years is probably owing to finite size sample variability in the POT analysis. Overall, we find that the westerly extreme wave heights at all locations both in the open North Atlantic and in the North Sea are correlated to the NAO, and stronger correlation is observed for locations in close proximity to the dominant storm tracks.

With the two predictor models, we produce two types of reconstructions of extreme wave climate at each location, assuming the large-scale trend longer than the period of available data is sufficiently captured by the NAO and other indices. Both reconstructions are robust in describing the short timescale fluctuations (less than 50 years), however, because the six-term model is able to explain the variability slightly better than the three-term model, the reconstruction from the modified six-term should be more reliable. Our reconstruction shows in general large decadal variability of extreme wave height for all locations. In particular, there is a recent heightened activity of extremes in the 1990s, which aligns with the strongly positive phase of the NAO, and there is a faster increase rate of the extremes for all locations in the period from 1960 to 1990, though there has to be uncertainty in our reconstructed results particularly the long-term trend prior to 1850s where Luterbacher's pressure reconstruction may not be as reliable as more recently. Reconstruction of mean wave climate in terms of annual mean *H*_s_ is also performed using similar methodology. Comparison with the historic extreme wave climate reveals that the variability in time of the mean, 99th percentile and extreme wave climates for locations in close proximity to the dominant storm tracks in the open North Atlantic are comparable, whereas the wave climates for the rest of the locations including the North Sea are rather different. From the reconstruction of 100 year return period, we observe that *H*_mp_∼2×*H*_sMax_, which implies that storms typically last longer than 3 h, perhaps 3× as long on average.

In this paper, we have concentrated on the 100 year wave (formally the 10^−2^ yr^−1^ probability level) and its decadal variability. This could be a realistic design criterion for a wave power installation, with a design life of a few decades and the consequences of failure being just financial. The design criteria for manned offshore platforms will be much more severe, with (much) longer return periods being used. In general, the historic extreme wave analysis reveals high levels of decadal variability for all locations, both in the open North Atlantic and also in the North Sea. Analysis of the autocorrelation structure suggests little predictability, unless forecasting of the NAO into the future can be improved. The recent results of Smith *et al.* [32] are encouraging in this regard.

## Supplementary Material

Additional evidence for decadal variability of extreme waves in the northeast Atlantic and North Sea

## Data accessibility

The climate indices are available from the NOAA/CPC at ftp://ftp.cpc.ncep.noaa.gov/wd52dg/data/indices/tele_index.nh. The reconstructed 500 mbar pressure maps by Luterbacher *et al*. [28] are available at https://www.ncdc.noaa.gov/paleo/pubs/luterbacher2002/luterbacher2002.html. Please contact the corresponding author for the proxy indices.
