# Supplementary material for: Decadal variability of extreme wave height representing storm severity in the northeast Atlantic and North Sea since the foundation of the Royal Society
Source: Proc Math Phys Eng Sci. 2016 Sep;472(2193):20160376. doi: 10.1098/rspa.2016.0376 (PMC5046986; doi:10.1098/rspa.2016.0376)
Supplement: Additional evidence for decadal variability of extreme waves in the northeast Atlantic and North Sea [file rspa20160376supp1.pdf]

# Supplementary Material for "Decadal variability of extreme wave height representing storm severity in the northeast Atlantic & North Sea since the foundation of the Royal Society"

by H. Santo, P. H. Taylor, and R. Gibson

## Contents of this file

1. Figures S1 to S7
2. Tables S1 to S3

## Introduction

This supporting information provides additional figures and tables for justification of the methods described in Section 2, 3, 4 and 5 in the paper.

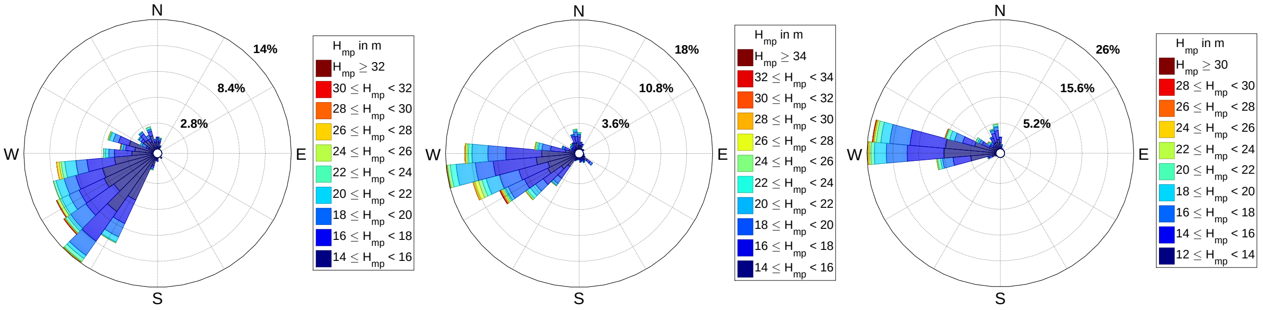


$(a)$

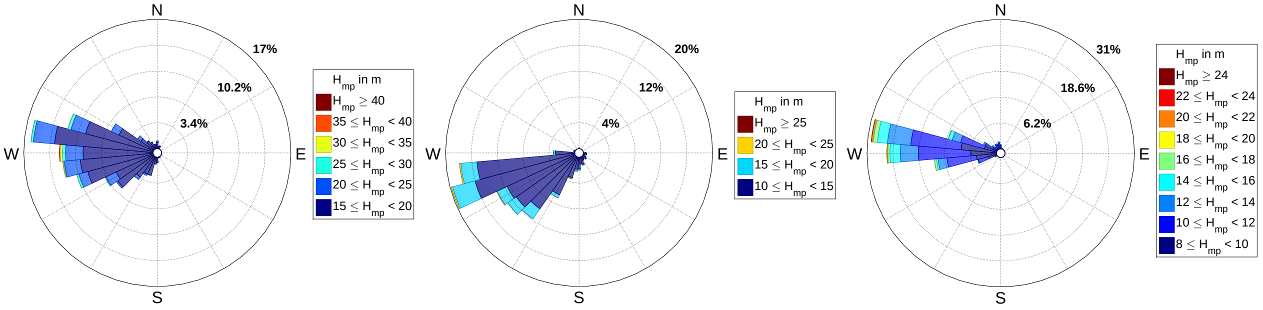


$(b)$

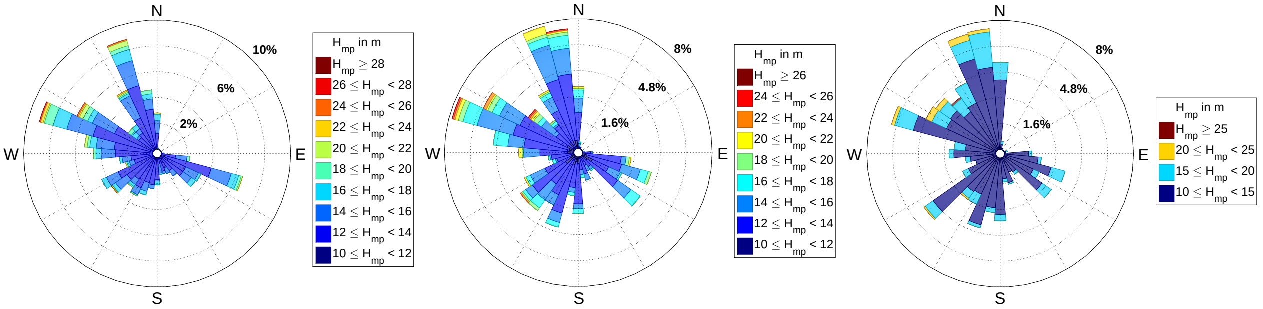


$(c)$

Figure S1: The largest 1000 storm severity distribution in terms of the most probable maximum individual wave height ($H_{mp}$). (a) For Haltenbanken, Schiehallion, and Orkney. (b) For Corrib, Kinsale and Cornwall. (c) For Draupner, Andrew and Forties. All from left to right. The range circles show the frequency (in %) of $H_{mp}$ occurrence normalised by the total occurrences for all directional bins of 10°

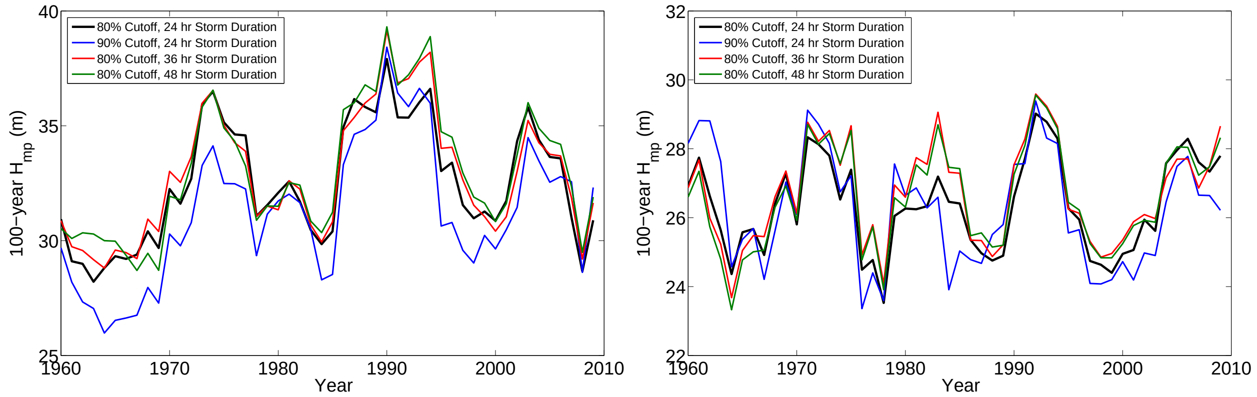


(a)

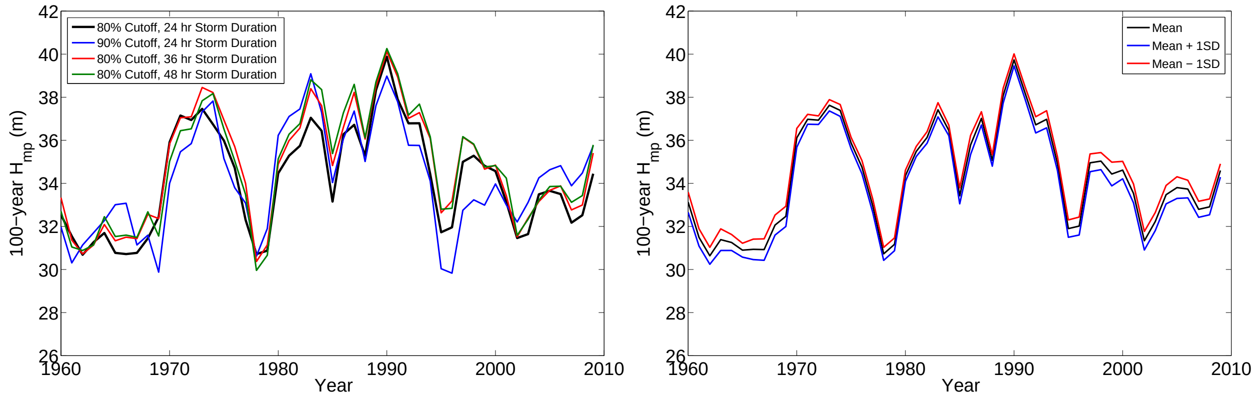


(b)

Figure S2: Comparison of the five-year sliding window of the 100-year $H_{mp}$ for different threshold values and storm durations imposed for storm-based identification. Similar temporal structures are recovered. All from left to right. (a) For Haltenbanken and Forties. (b) For Schiehallion (left). Also shown on the right is the internal variability by repeating the same analysis 100 times for Schiehallion for 80% threshold and 24 hour storm duration. Hence, some of the variability observed from different threshold values and storm durations are due to internal sample variability.

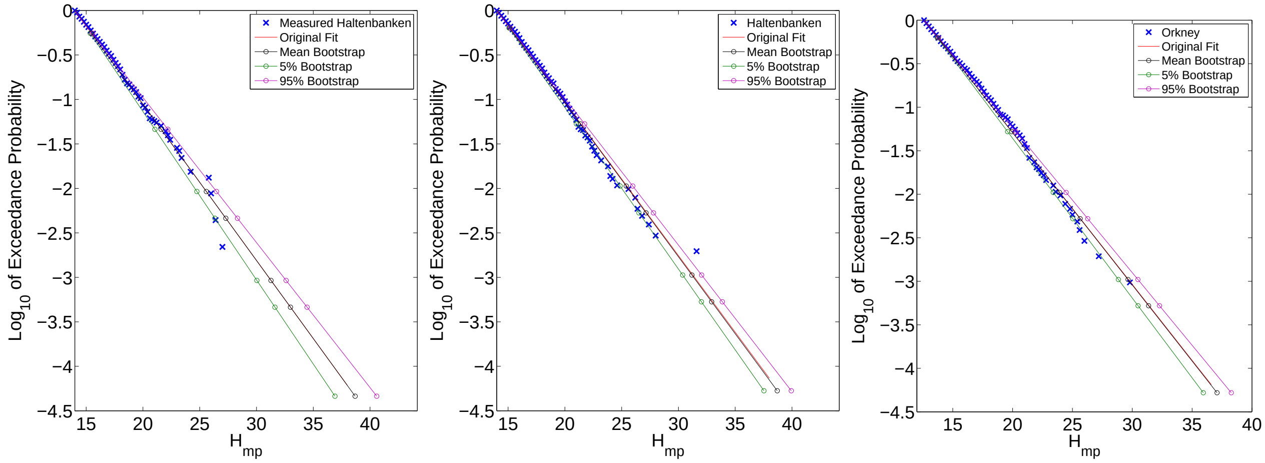


$(a)$

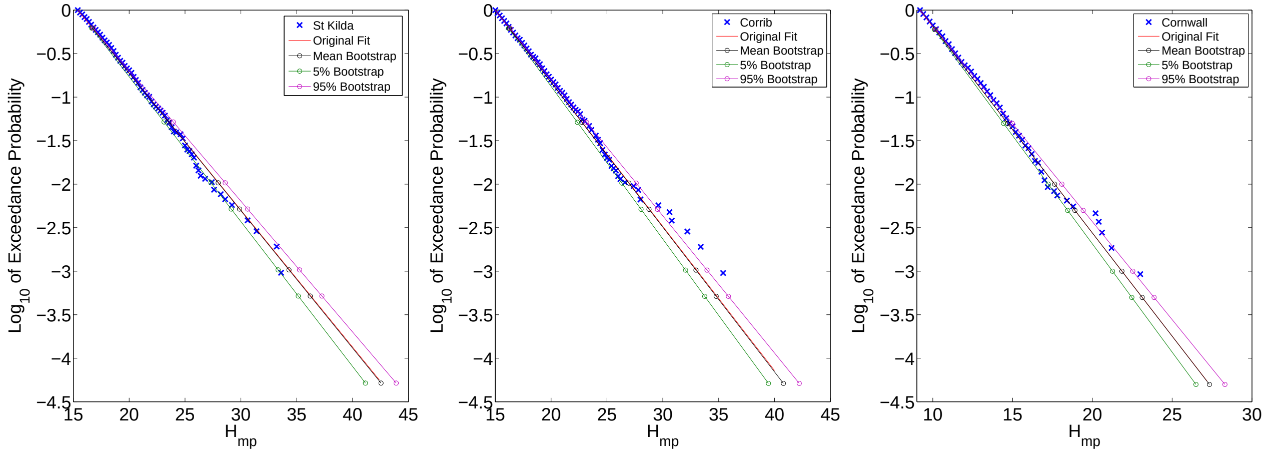


$(b)$

Figure S3: Trumpet plot from the exponential fit with parametric bootstrap for threshold $N \sim 1000$ largest storms. The circles from bottom up correspond to $H_{mp}$ with a return period of 1000 years, 100 years, 50 years, 10 years, 5 years, 1 year and 1 month, respectively. (a) For measured Haltenbanken, Haltenbanken, and Orkney. (b) For St Kilda, Corrib and Cornwall. All from left to right. All trumpet plots are based on 54 years of data, except measured Haltenbanken which is based on 22 years of data.

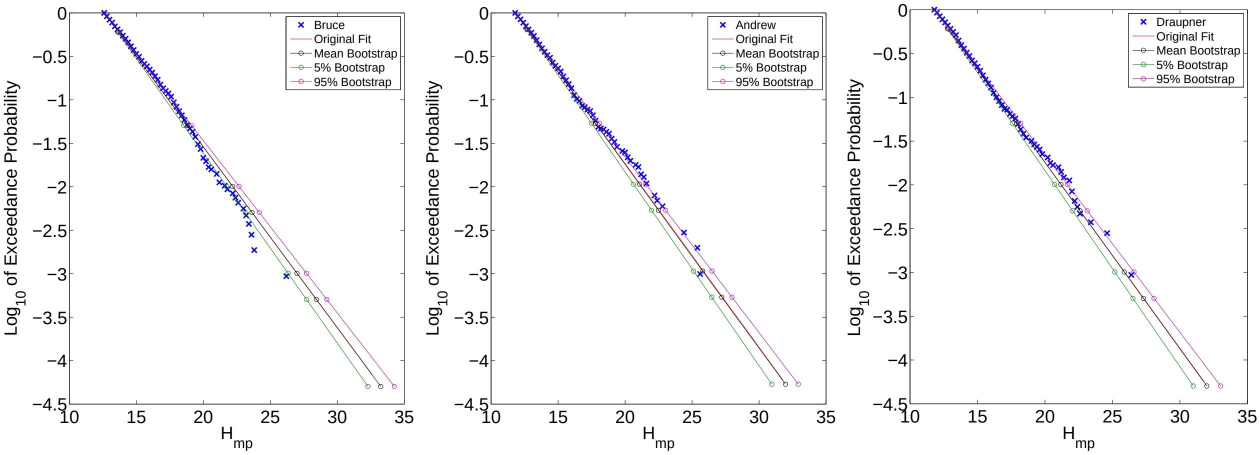


(*a*)

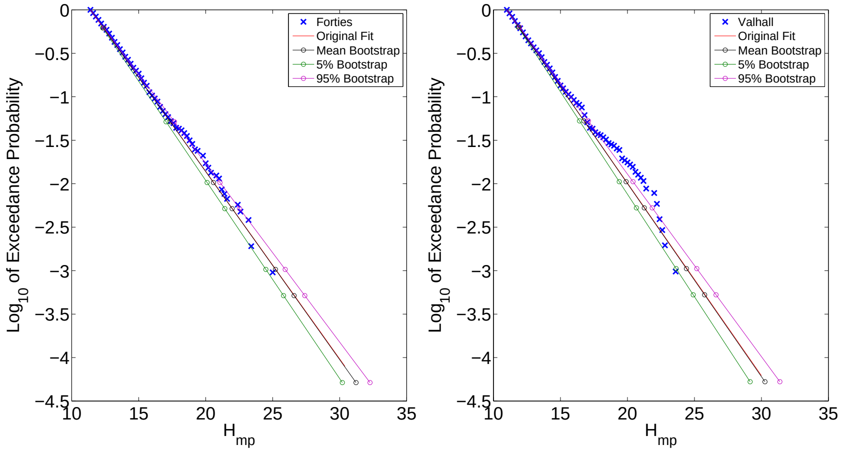


(*b*)

Figure S4: Trumpet plot from the exponential fit with parametric bootstrap for threshold $N \sim 1000$ largest storms. The circles from bottom up correspond to $H_{mp}$ with a return period of 1000 years, 100 years, 50 years, 10 years, 5 years, 1 year and 1 month, respectively. (a) For Bruce, Draupner and Andrew. (b) For Forties and Valhall. All from left to right.

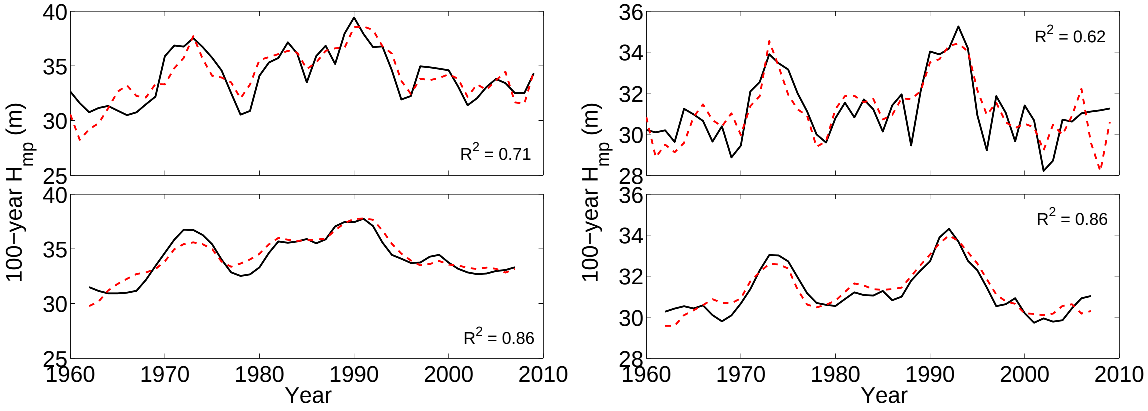


$(a)$

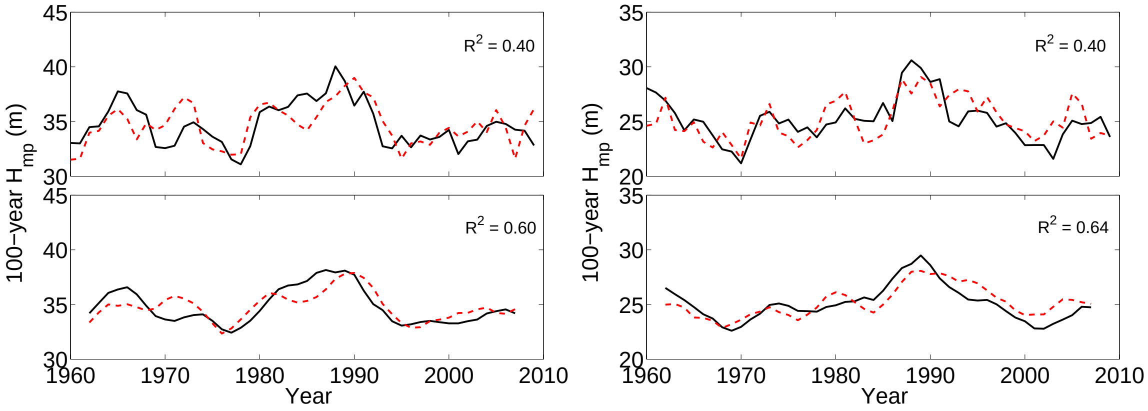


$(b)$

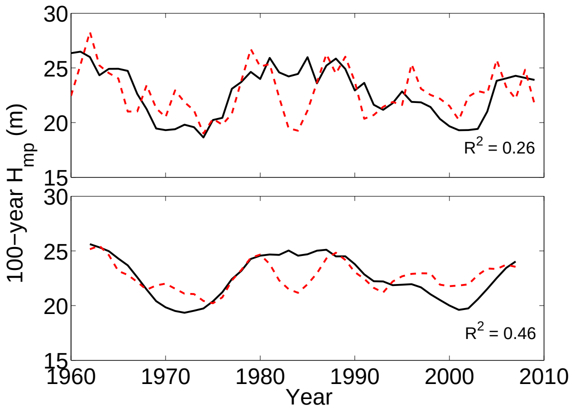


$(c)$

Figure S5: Comparison in terms of 1 in 100 year $H_{mp}$ (solid lines) five-year sliding window and the teleconnection-based prediction (dashed lines) using the 6-term predictor model. (a) For Schiehallion and Orkney. (b) For Corrib and Kinsale Head. (c) For Cornwall. All from left to right. Top figure shows five-year sliding window, bottom figure with an imposed five-year moving average.

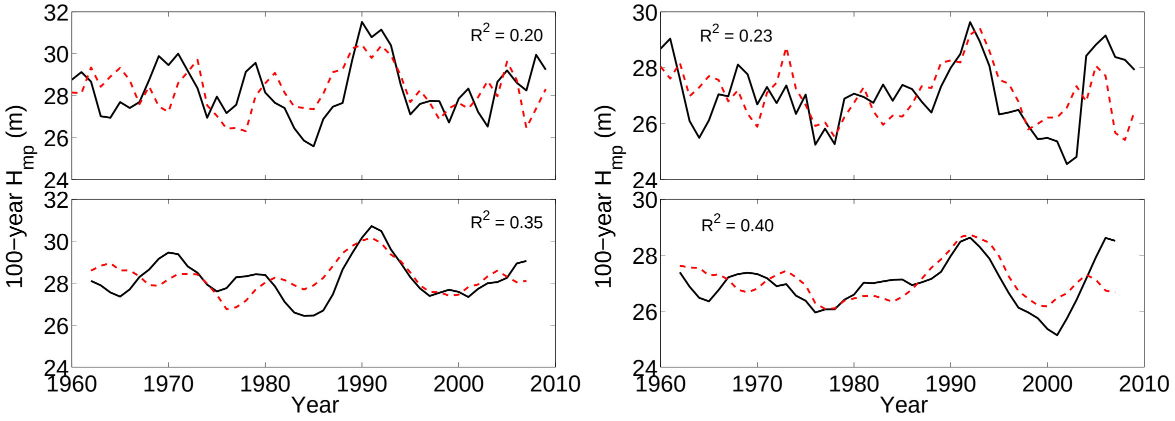


(*a*)

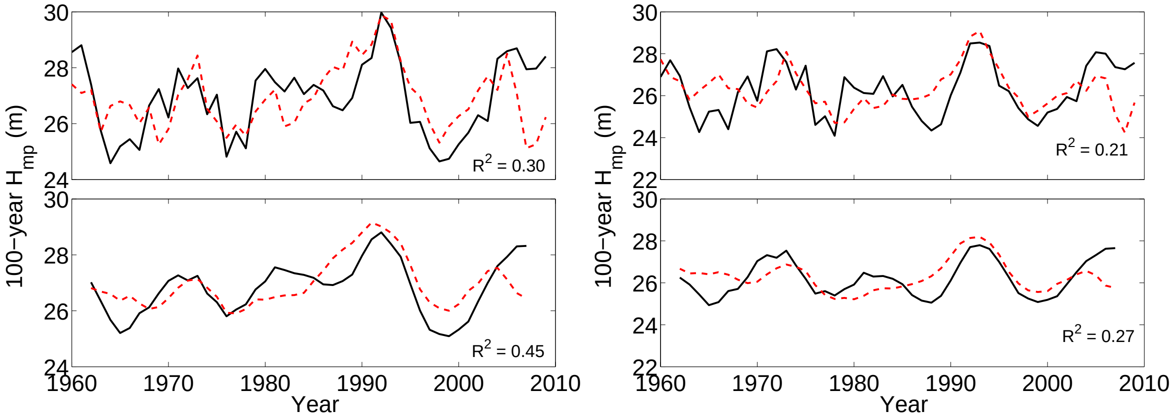


(*b*)

Figure S6: Comparison in terms of 1 in 100 year $H_{mp}$ (solid lines) five-year sliding window and the teleconnection-based prediction (dashed lines) using the 6-term predictor model. (a) For Bruce and Draupner. (b) For Andrew and Forties. All from left to right. Top figure shows five-year sliding window, bottom figure with an imposed five-year moving average.

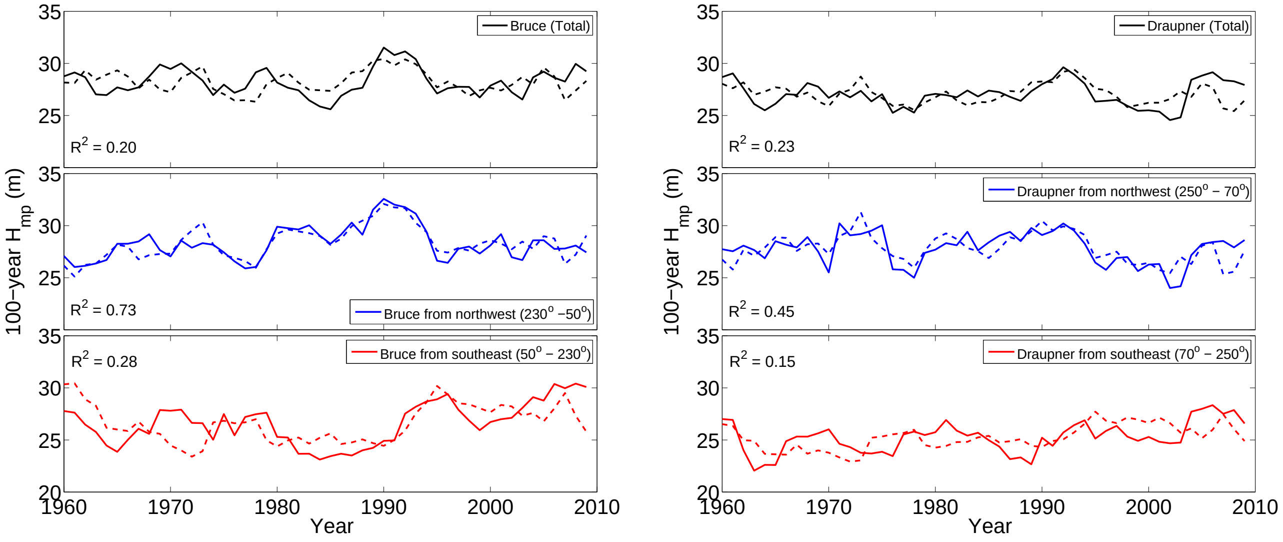


(*a*)

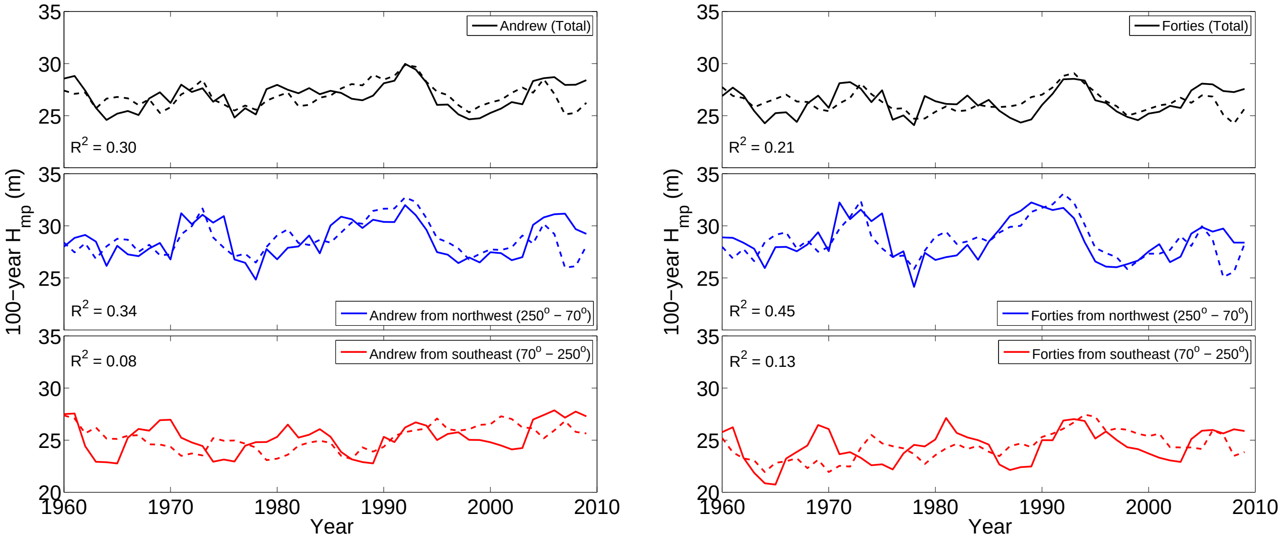


(*b*)

Figure S7: Comparison of the 1 in 100 year $H_{mp}$ (solid lines) and the teleconnection-based prediction (dashed lines) using the 6-term predictor model for the locations in the North Sea with angle partitioning. (a). For Bruce and Draupner. (b) For Andrew and Forties. All from left to right.

Table S1: Summary of the results of the long-return period exponential fits to $H_{mp}$ for different threshold values and storm durations imposed for storm-based identification.

| Location | 80% cutoff & 24 hr storm duration | 90% cutoff & 24 hr storm duration | 80% cutoff & 36 hr storm duration | 80% cutoff & 48 hr storm duration |
|---|---|---|---|---|
| Haltenbanken | | | | |
| $H_{mp-100}$ (m) | 33.0 | 32.1 | 33.3 | 33.4 |
| $H_{mp-1000}$ (m) | 38.8 | 37.3 | 39.2 | 39.4 |
| Schiehallion | | | | |
| $H_{mp-100}$ (m) | 34.7 | 34.7 | 35.2 | 35.3 |
| $H_{mp-1000}$ (m) | 40.8 | 40.5 | 41.4 | 41.5 |
| Forties | | | | |
| $H_{mp-100}$ (m) | 26.6 | 26.3 | 26.9 | 26.8 |
| $H_{mp-1000}$ (m) | 31.3 | 30.7 | 31.7 | 31.5 |

Table S2: Summary of the strength of the non-dimensionalised constants from the correlation with the teleconnections for all locations.

| Location | 3-term model $b_1/c_1/d_1$ | 6-term model $(b_1,b_2)/(c_1,c_2)/(d_1,d_2)$ |
|---|---|---|
| For the open North Atlantic locations: | | |
| 1. Haltenbanken | 0.27/-0.07/0.11 | (0.25,0.08)/(-0.09,-0.03)/(0.07,0.16) |
| 2. Schiehallion | 0.23/-0.11/0.01 | (0.20,-0.06)/(-0.09,-0.05)/(-0.01,-0.09) |
| 3. Orkney | 0.16/-0.12/0.02 | (0.15,0.05)/(-0.13,-0.07)/(-0.01,-0.02) |
| 4. St Kilda | 0.22/-0.05/0.12 | (0.21,-0.11)/(-0.02,0.05)/(0.14,-0.09) |
| 5. Corrib | 0.12/-0.15/-0.20 | (0.11,-0.12)/(-0.09,0.13)/(-0.10,-0.05) |
| 6. Kinsale Head | 0.11/-0.13/-0.36 | (0.12,0.10)/(-0.14,0.09)/(-0.29,0.29) |
| 7. Cornwall | 0.23/-0.03/-0.67 | (-0.13,0.03)/(-0.04,0.23)/(-0.44,0.43) |
| For the North Sea locations: | | |
| 8. Bruce | 0.08/-0.12/-0.14 | (0.10,0.01)/(-0.11,0.11)/(-0.07,0.04) |
| 9. Draupner | 0.09/-0.12/-0.05 | (0.10,0.07)/(-0.11,0.06)/(-0.02,0.07) |
| 10. Andrew | 0.14/-0.09/0.01 | (0.15,0.05)/(-0.08,0.11)/(0.05,0.10) |
| 11. Forties | 0.13/-0.13/0.05 | (0.12,0.09)/(-0.10,0.04)/(0.07,0.03) |
| 12. Valhall | 0.15/-0.22/-0.21 | (0.16,0.12)/(-0.23,-0.01)/(-0.18,0.01) |

Table S3: Summary of the strength of the non-dimensionalised constants from the correlation with the teleconnections for the North Sea locations with angle partitioning.

| Location | 3-term model $b_1/c_1/d_1$ | 6-term model $(b_1,b_2)/(c_1,c_2)/(d_1,d_2)$ |
|---|---|---|
| Bruce | | |
| Omnidirectional | 0.08/-0.12/-0.14 | (0.10,0.01)/(-0.11,0.11)/(-0.07,0.04) |
| Northwest | 0.16/-0.10/-0.08 | (0.17,-0.05)/(-0.09,0.05)/(-0.05,-0.07) |
| Southeast | -0.21/0.22/0.00 | (-0.11,0.21)/(0.09,-0.06)/(-0.01,0.05) |
| Draupner | | |
| Omnidirectional | 0.09/-0.12/-0.06 | (0.10,0.07)/(-0.11,0.06)/(-0.02,0.07) |
| Northwest | 0.13/-0.18/-0.10 | (0.13,-0.01)/(-0.18,0.01)/(-0.09,0.02) |
| Southeast | -0.06/0.17/0.04 | (-0.05,0.11)/(0.12,-0.07)/(0.01,0.01) |
| Andrew | | |
| Omnidirectional | 0.14/-0.09/-0.01 | (0.15,0.05)/(-0.08,0.11)/(0.05,0.10) |
| Northwest | 0.18/-0.14/-0.03 | (0.20,0.03)/(-0.14,0.08)/(0.00,0.07) |
| Southeast | -0.11/0.17/0.08 | (-0.03,-0.10)/(0.07,-0.01)/(0.07,-0.11) |
| Forties | | |
| Omnidirectional | 0.12/-0.10/-0.07 | (0.12,0.09)/(-0.10,0.04)/(0.07,0.03) |
| Northwest | 0.21/-0.18/0.01 | (0.23,-0.01)/(-0.17,0.11)/(0.06,0.03) |
| Southeast | 0.09/0.06/-0.01 | (0.06,0.15)/(0.01,-0.13)/(0.05,-0.02) |
